# Ten Years of *Preventing Chronic Disease*

**DOI:** 10.5888/pcd10.120245

**Published:** 2013-01-03

**Authors:** Lynne Wilcox

Ten years are a blink. Ten years are a lifetime. It has been 10 years since the first issue of *Preventing Chronic Disease: Public Health Research, Practice, and Policy (PCD)*. In our introductory editorial, we defined our primary audience as researchers in chronic disease prevention and intervention and health practitioners responsible for reducing the effects of chronic conditions and improving population health (1). We also identified our goals: to promote dialogue between researchers and practitioners, describe interdisciplinary and multisectorial approaches to chronic diseases, and present new theories and concepts of research and practice.

The mission of *PCD* is “to promote the open exchange of information and knowledge among researchers, practitioners, policy makers, and others who strive to improve the health of the public through chronic disease prevention” (2). Public health researchers told us that other journals did not understand or publish prevention research. Practitioners told us they seldom found material in research journals that was relevant to their work. Yet both research findings and practical demonstrations are essential for evidence-based public health policy and programs. *PCD* was designed to address that gap.

We initiated action to address those goals in the inaugural issue. It included articles on multiple health topics, including nutrition, osteoporosis, dementia, breast and cervical cancer screening, and social determinants of health. The study populations included homebound seniors, school children, and racial/ethnic minority populations; the research methods included qualitative semistructured interviews, telephone interviews, public health surveillance, and a dementia registry. The inaugural issue also addressed the role of the community in public health in an article and a book review on community-based participatory research; other articles described population-based interventions and the use of public health law.

The inaugural issue included a video on a community-based tobacco use control program. *PCD*’s premise was that an online journal should go beyond traditional text and take advantage of the ability to offer a range of audiovisual materials. In future issues, *PCD* would include videos on breast and cervical cancer screening in Navajo women, the African American Health Coalition in Oregon, a preview from the Public Broadcasting Service health series *Unnatural Causes: Is Inequality Making Us Sick?*, a school-based health education program in São Paulo, Brazil, and many others.

We also wanted *PCD* to be an international journal. Our first issue to include Spanish-language articles was January 2005, and it focused on the Border Health Strategic Initiative (*Border Health* *¡SI!*) on the US–Mexico border in Arizona. Today, most *PCD* articles include Spanish translations.

In that 10-year blink, how has *PCD* evolved? A perusal of journal content between December 2011 and July 2012 finds articles on not only subjects of ongoing public health interest such as social determinants, nutrition, breast health, diabetes, and surveillance but also topics such as health financing, veterans’ health, and health informatics. International health is represented by reports from Mexico, Australia, Canada, Jordan, Ethiopia, and Grenada.

*PCD* is now published continually, provides new media opportunities (eg, Twitter, Facebook, RSS feeds, embedding, e-cards, a *PCD* app), and offers continuing medical education credits. The number, breadth, and quality of the articles have increased. The journal supports an annual student paper competition and provides podcasts of selected authors discussing their work. In addition, the journal has added a fourth goal to the original 3: encouraging multisectoral partnerships that engage communities (2).

Ten years are a lifetime. The classic life-cycle metaphor can be used to describe the growth and development of *PCD* to date: gestation, birth, infancy, childhood, and adolescence. The journal is well positioned to move on to young adulthood and maturity. Happily, a journal need not experience senescence or death, and we can look for increasing quality, influence, and readership in the future.

Ten years ago, my first editorial spoke of chronic diseases in my parents’ generation. Today, all of that generation of my family is gone, and among my own generation others have been lost to chronic diseases. Let us hope our work will enhance the health of our children — they, too, have a lifetime, and we will be gone in a blink.
